# Exploring the Role of *SIRT1* Polymorphisms in Colorectal Cancer Risk: A Case–Control Study

**DOI:** 10.3390/jcm14113912

**Published:** 2025-06-02

**Authors:** Justyna Klusek, Piotr Lewitowicz, Grażyna Nowak-Starz, Bartosz Witczak, Ruslan Oblap, Dorota Kozieł, Anna Nasierowska-Guttmejer, Jolanta Klusek, Artur Jóźwik, Tomasz Rogula, Kamila Kocańda, Stanisław Głuszek

**Affiliations:** 1 Collegium Medicum, Jan Kochanowski University, 25-369 Kielce, Poland; 2 Faculty of Medicine, Lazarski University, 02-662 Warszawa, Poland; 3 Collegium Medicum, Społeczna Akademia Nauk, 90-113 Lodz, Poland; 4 Polish Academy of Sciences, Institute of Genetics and Animal Biotechnology, 05-552 Jastrzębiec, Poland; 5 Świętokrzyskie Centrum Onkologii, 25-734 Kielce, Poland

**Keywords:** colorectal cancer (CRC), *SIRT1*, single-nucleotide polymorphism (SNP)

## Abstract

**Background:** Colorectal cancer (CRC), the most common malignancy of the gastrointestinal tract, is the second leading cause of cancer-related deaths worldwide. In this context, investigating low-penetrance gene variants associated with the increased risk of CRC represents a novel and crucial approach to enhancing prevention strategies and clinical surveillance. By focusing on these genetic variants, there is potential for more accurate prediction of individual CRC risk, which could contribute to the refinement of current screening and prophylactic programs. The aim of this case–control study was to explore the association between SIRT1 polymorphisms and CRC risk. **Methods:** We analyzed three SNPs—rs12778366 (T/C), rs3758391 (C/T), and rs7895833 (A/G)—in the promoter region of the SIRT1 gene, which may influence SIRT1 expression and thus play a role in cancer development. Our study included 200 patients with colorectal adenocarcinoma and 115 controls. Genomic DNA was extracted from blood samples, and SIRT1 SNP analysis was performed using the qPCR method and endpoint genotyping. **Results:** Univariate regression analysis revealed a slightly increased risk of developing CRC in individuals with minor alleles of the analyzed polymorphisms; however, the observed differences were not statistically significant. **Conclusions:** Although our findings did not reveal statistically significant differences in SIRT1 gene polymorphism frequencies between the CRC group and the control group, we observed a tendency that suggests further investigation in larger cohorts is warranted. This research underscores the importance of understanding low-penetrance genetic factors in CRC, highlighting their potential to inform more personalized and effective prevention strategies.

## 1. Introduction

Colorectal cancer (CRC), the most common cancer of the gastrointestinal tract, is the second leading cause of cancer-related fatality worldwide [1]. Based on available statistics, a 30% higher incidence rate is reported in men versus women [2]. In developed countries, the average lifetime risk of CRC is about one in twenty-three (4.3%) for men and one in twenty-five (4.0%) for women [3]. This sex imbalance is due to the influence of sex hormones and lifestyle differences. However, an estimated 12–35% risk of colorectal cancer incidence in both sexes is attributed to genetic factors [2]. This includes not only rare high-penetrance mutations, such as HNPCC (hereditary non-polyposis colorectal cancer) or FAP (familial adenomatous polyposis coli), which elevate the risk for hereditary syndromes, but also common polymorphisms. Discovering these common variants associated with an elevated risk of CRC will enable a more accurate prediction of an individual’s risk, thereby improving prevention strategies and clinical surveillance [4]. Among these low-penetrance genes that may play a significant role in cancer risk is *SIRT1*.

*SIRT1* is a member of the sirtuin gene family, proteins that modulate gene expression in response to the cell’s energy status [5,6]. It is found both in the nucleus and cytoplasm, and due to its involvement in key cellular processes such as the regulation of the cell cycle, differentiation, aging, apoptosis, and mitochondrial metabolism, it has become the most extensively investigated member of the sirtuin family [7]. As it is a metabolic-sensitive protein, *SIRT1*’s expression is upregulated during fasting or nutrient deprivation [8]. The *SIRT1* gene product, by deacetylating histones, transcription factors, and transcription cofactors, regulates diverse cellular processes such as aging, apoptosis, autophagy, immune response, and DNA repair [5,6]. Its role in carcinogenesis is therefore highly likely but remains controversial due to these multiple functions. Evidence suggests that *SIRT1* may function as both a tumor suppressor and a tumor promoter, depending on the context [9,10]. For example, collaborating with histone deacetylase 1 (HDAC1) improves genetic stability [11] and promotes DNA repair upon genotoxic stress [12]. On the other hand, via the deacetylation of the p53 protein, *SIRT1* decreases p53 activity and acts anti-apoptotically, promoting tumor development [13,14], and has been found to be tumorigenic in various human cancers [12]. *SIRT1* may function in a context-dependent manner to exert tumor-promoting or suppressing qualities [5,14].

Increasing evidence suggests the significance of *SIRT1* gene polymorphisms in modulating colorectal cancer (CRC) risk. According to Simons (2018) [5], *SIRT1* gene polymorphism influences the risk of colon cancer in women, with the rs12778366 CC genotype being associated with a decreased risk of colorectal and colon cancer in this population, whereas no such effect has been observed in males. Moreover, interactions between *SIRT1* variants and energy balance-related factors were identified. The influence of the *SIRT1* polymorphism on the risk of developing CRC, specifically MSI CRC (microsatellite instability-positive colorectal cancer), was also demonstrated by Hriz (2020) [9]. These observations justify the present case–control study, which aims to investigate the association between specific *SIRT1* polymorphisms and colorectal cancer risk. We studied three SNPs (single-nucleotide polymorphisms)—rs12778366 (T/C), rs3758391 (C/T), and rs7895833 (A/G)—of the *SIRT1* gene promoter region, which may modulate *SIRT1* expression and play a potential role in carcinogenesis. As described in our previous study [15], colorectal cancer (CRC) represents a heterogeneous group of malignancies in which the molecular and clinical characteristics vary significantly depending on tumor location. Given these differences, including distinct embryological origins, mutational profiles, histopathological features, progression patterns, and survival outcomes, the stratification of CRC cases according to anatomical location (right-sided colon, left-sided colon, and rectum) was applied in the current study. This approach allows for a more nuanced analysis of the potential genetic associations within more biologically uniform subgroups.

## 2. Materials and Methods

### 2.1. Population Sample

This study was conducted at two clinical centers in Kielce, Poland: the Kielce Provincial Hospital and the Holy Cross Cancer Centre. The inclusion criteria were age over 18, documented informed consent, and completion of a colonoscopy examination. Exclusion criteria included known hereditary colorectal cancer syndromes (such as familial adenomatous polyposis [FAP] and hereditary nonpolyposis colorectal cancer [HNPCC]), a family history of colorectal cancer, the presence of colonic polyps, and pregnancy. Our study included 199 patients with colorectal adenocarcinoma confirmed by pathological diagnosis of specimens collected during colonoscopy or surgery (a total of 200 patients were initially recruited; however, one had to be excluded due to the absence of precise tumor localization in the histopathological report). The control group consisted of 120 patients without colorectal cancer or polyps as confirmed by an endoscopic and/or histopathological examination. The research protocol was approved on 3 June 2013, by the local Bioethics Commission (No. 5/2013) with the annotation on 4 November 2022. All procedures performed in the study followed the institution’s ethical standards, the Helsinki Declaration, and its later amendments. All patients signed written consent forms for genetic testing. Clinical data and blood samples were collected (test tubes with EDTA provided by Sarsted, Warszawska 25, 05-082 Stare Babice, Poland). Coded samples were frozen at −80 °C for genetic testing.

### 2.2. Genotyping

Peripheral blood leukocytes were the material used for genetic testing. The genomic DNA was extracted from blood samples using the automatic nucleic acid extractor and genomic DNA whole blood kit (Magcore^®^, RBC BioScience, New Taipei, Taiwan). The purity and concentration of the isolated DNA were evaluated spectrophotometrically at 260 nm and 280 nm (Denovix, DS-11, Wilmington, DE 19810, USA). Analysis of the *SIRT1* SNPs rs12778366, rs3758391, and rs7895833 was performed using the TaqMan^®^ genotyping assays (Applied Biosystems, Foster City, CA, USA), according to the manufacturer’s instructions and the qPCR method and endpoint genotyping. PCR amplification using ≈10 ng of genomic DNA was conducted with an initial step of 95 °C for 10 min, followed by 50 cycles of 95 °C for 15 s (denaturation step) and 60 °C for 60 s (annealing and elongation step).

### 2.3. Statistical Analysis

The sample size was calculated under the assumption of a dominant genetic model, with an expected odds ratio (OR) of 2.0 and a minor allele frequency (MAF) of 20%. To achieve a statistical power of 80% at a two-sided significance level of 5% (α = 0.05), the required number of participants was estimated at 223 in the CRC group and 114 in the control group, maintaining a 2:1 case-to-control ratio. Hardy–Weinberg equilibrium was tested by a chi-square goodness-of-fit test. Continuous data were described by means and standard deviations, whereas categorical data were summarized by frequencies and percentages. Group comparisons were performed using the chi-square test for categorical variables and the t-test for continuous variables. Logistic regression models were used to investigate the association between CRC and *SIRT1* gene polymorphism. A non-adjusted (crude) as well as adjusted (for sex, age, and BMI) odds ratios (OR) with 95% confidence intervals were calculated to assess the strength of the aforementioned associations.

A two-tailed *p*-value <0.05 was considered statistically significant. All statistical analyses were performed using the R software package version 4.0.3.

## 3. Results

The demographic characteristics of the study population, including tumor localization, are presented in Table 1. Among patients with cancer, rectal cancer was the most commonly represented (42.7% of the study group). The control group was well matched to the study group in terms of age and BMI.

The observed predominance of males in the CRC group is consistent with global epidemiological data indicating a higher incidence of colorectal cancer in men. Demographic analysis stratified by tumor location also revealed that right-sided colorectal cancer tends to occur in older individuals (*p* = 0.0045). However, as demonstrated by subsequent multivariable analysis, neither sex nor age was found to be a confounding factor in this study, and neither had a significant impact on the genetic associations under investigation. Nevertheless, to ensure the robustness of the results, additional analyses stratified by sex were performed, and their results are presented in Supplementary Materials (Supplementary Tables S1 and S2). These analyses did not reveal any sex-specific differences in the association between *SIRT1* polymorphisms and colorectal cancer occurrence.

The distribution of *SIRT1* gene polymorphisms in patients with CRC and controls was analyzed using the dominant genetic model, as shown in Table 1. The minor alleles of SNPs rs12778366 and rs3758391 were more frequently observed in individuals with cancer, whereas the minor allele of rs7895833 was more prevalent in the control group. However, none of these differences reached statistical significance.

The associations between *SIRT1* gene polymorphisms and BMI were also analyzed in this study. The results are shown in Table 2.

BMI differences by *SIRT1* genotype were generally not statistically significant in either the control or CRC groups, except for rs3758391 in the control group (*p* = 0.0327), where individuals carrying the minor allele had a lower mean BMI compared to those with the CC genotype. No significant associations between BMI and *SIRT1* genotypes were observed among patients with CRC.

The results of univariate and multivariable regression analyses are presented in Table 3.

For all three analyzed *SIRT1* gene polymorphisms, no statistically significant association with colorectal cancer risk was observed, either in unadjusted models or in models adjusted for potential confounding factors such as sex, age, and BMI. Although some variants showed a tendency toward increased risk (e.g., rs12778366 with OR >1), the lack of statistical significance suggests that any potential effect is likely modest or may require a larger sample size to be detected.

Subsequently, CRC cases were stratified according to anatomical subsite—right-sided colon, left-sided colon, and rectum—for further analysis. Although no statistically significant associations between *SIRT1* genotype and cancer risk were observed for any specific tumor location, even after adjustment for sex, age, and BMI, the previously noted tendency may persist. It is possible that subgroup sample sizes were too small to reveal potential differences after the stratification. The detailed results of this analysis are presented in the Supplementary Materials (Supplementary Tables S3–S5).

## 4. Discussion

*SIRT1* is recognized as a longevity-associated gene that protects cells from oxidative and genotoxic stress by deacetylating a variety of target proteins, including p53 and members of the FOXO family. As a result, cell survival and DNA repair are enhanced. Emerging evidence also highlights its significant role in regulating glucose homeostasis and lipid metabolism. Moreover, *SIRT1* is involved in essential cellular responses to oxidative stress and inflammation by enhancing the transcriptional activity of downstream genes [16]. Thus, it may play a significant role in human pathophysiology, and some evidence suggests an important role in diverse diseases, including cancer [17]. *SIRT1* may play multiple—and potentially opposing—roles in cancer, either contributing to tumorigenesis or protecting cells from it. Its role in cancer occurrence, progression, or survival remains controversial, as *SIRT1* has been reported to exert both oncogenic and tumor-suppressive effects [18]. In some pathophysiological conditions, including tumor progression, *SIRT1* seemed to activate protective signaling pathways controlled by AKT (serine/threonine-specific protein kinases) and PDK1 (phosphoinositide-dependent protein kinase 1) [19]. On the other hand, the meta-analyses carried out by Wu et al. in 2017 and Sun et al. in 2019 revealed that overexpression of *SIRT1* indicates a poor prognosis for patients with various cancers [20,21]. Therefore, the role of *SIRT1* in tumorigenesis might depend on the temporal and spatial distribution of upstream regulators and downstream targets [10]. The analyzed *SIRT1* gene polymorphisms rs12778366 (T/C), rs3758391 (C/T), and rs7895833 (A/G) are located in the promoter region of the gene and may therefore affect its expression [5,22,23].

It is known that heterozygotes and homozygotes for the C allele of rs12778366 show higher *SIRT1* expression than their wild-type counterparts [5]. In our study, a higher frequency of the rs12778366 minor C allele was observed in patients with CRC, especially right-sided colorectal cancer (Table 2). However, the difference was statistically insignificant. Consistently, other case–control studies investigating this polymorphism in cancer have not reported statistically significant associations [10,24]. In contrast, Simons (2018) found that the minor C allele of rs12778366 was associated with a reduced risk of colorectal cancer, but in a sex-dependent manner—this association was observed only in women [5]. Our sex-stratified analysis did not confirm this finding (Supplementary Table S1). According to Figarska et al. (2013), the protective effect of this allele appears to be sex-independent, as Dutch carriers exhibited a significantly reduced mortality risk compared to wild-type individuals in the general population—an effect observed in both males and females, including smokers and those who are overweight or have obesity [25]. These findings suggest that higher expression may exert a protective effect against the development of CRC; however, this was not observed in our study. According to Ren et al. (2017), reduced *SIRT1* expression is associated with the increased proliferation of colorectal adenocarcinoma cells, tumor development, and poor prognosis in patients [26]. This association has been attributed to the proposed ability of *SIRT1* to upregulate c-Myc, thereby potentially promoting malignant behavior and unfavorable clinical outcomes [18]. Therefore, the absence of this effect in our study population may result from polymorphisms in the target genes.

The *SIRT1* single-nucleotide polymorphism rs3758391 (C/T) has been shown to reduce gene expression in vitro [8]. Moreover, due to its location at the p53-binding site of the *SIRT1* gene, this polymorphism may disrupt the p53-binding sequence [17]. Reduced gene expression, along with impaired interaction with the tumor suppressor p53, may negatively affect cellular mechanisms involved in tumor suppression. Kan and colleagues demonstrated that the *SIRT1* rs3758391 polymorphism is associated with both the risk and survival outcomes of diffuse large B-cell lymphoma (DLBCL) in the Chinese Han population. The TT genotype and the T allele were significantly more frequently observed in DLBCL patients compared to healthy controls [17]. Vaiciulis et al. (2022) reported that the TT genotype of the *SIRT1* rs3758391 variant is linked to an elevated risk of developing laryngeal squamous cell carcinoma [27]. Similarly, a study conducted on a population of individuals with breast cancer demonstrated an association between the T allele of rs3758391 and both an increased risk and poorer prognosis of malignancy. In this investigation of an Egyptian population, the TT genotype and T allele were significantly more prevalent among patients with breast cancer compared to healthy controls [22]. In contrast, our study did not demonstrate a significant relationship between rs3758391 and colorectal cancer (CRC). This finding may be influenced by factors not assessed in our analysis, such as dietary habits, especially since this polymorphism has been reported to modulate the expression of nutrient-responsive genes regulated by *SIRT1* [8].

*SIRT1* polymorphism rs7895833 (A/G) is also located in the gene promoter, and its base sequence is TTGACT, which has been proven to be a W-box-like element of the promoter, which may affect the binding affinity of transcription factors and subsequently alter gene expression levels [16]. There is growing evidence suggesting a potential role of this polymorphism in cancer susceptibility. It was demonstrated that the *SIRT1* rs7895833 G/G genotype was associated with an approximately 13-fold increased risk of developing active pituitary adenoma compared to carriers of the A allele (A/A or A/G genotypes) [28]. Our results did not confirm an increased cancer risk in the presence of the G allele.

Beyond oncology outcomes, several studies have reported associations between this polymorphism and BMI and metabolic syndrome among diverse populations. In the Rotterdam Study, rs7895833 had a significant association with BMI at baseline, with an allele-dose effect (G allele was associated with lower BMI) [29]. The *SIRT1* rs7895833 allele A has been associated with an increased risk of metabolic syndrome in a Chinese Han population [16]. Consistent with this, in a Japanese population study, A allele carriers demonstrated a significantly elevated risk of obesity [30]. In another study, Mexican males carrying the G allele of rs7895833 demonstrated slightly lower BMI levels [31]. Given that elevated BMI is a well-established risk factor for colorectal cancer, the potential influence of *SIRT1* polymorphisms on body weight regulation may be an important intermediate factor in understanding genotype–phenotype–disease associations. In our Caucasian population, we did not observe any such association for rs7895833. However, we did observe statistically significant differences in mean BMI among individuals carrying the minor T allele of rs3758391 (Table 2). Interestingly, the observed association was restricted to controls and did not persist in individuals with CRC, which might suggest that the presence of cancer itself alters or obscures such genotype-related patterns. This finding strengthens our rationale that the current analysis should be considered preliminary and suggests that further investigations involving a larger study population may be justified.

Given the well-established heterogeneity of colorectal cancer based on tumor location—with distinct embryological origins, molecular pathways, histological features, and metastatic patterns—the assessment of *SIRT1* polymorphisms in relation to tumor site may provide additional insights into their role in CRC pathogenesis. Right- and left-sided colorectal cancers, as well as rectal cancers, differ not only in clinical behavior and prognosis but also in genetic background [2,15]. Therefore, analyzing the association between *SIRT1* variants and tumor location may help clarify their potential site-specific contribution to colorectal carcinogenesis. Nevertheless, in our study, we did not observe any significant association between this polymorphism and tumor development at any of the analyzed locations, probably due to the limited sample size in each subgroup after stratification by tumor site.

Several limitations should be considered when interpreting the results of this study. First, the relatively small sample size may have limited the statistical power to detect significant associations between *SIRT1* polymorphisms and colorectal cancer risk. As a result, subtle genetic effects could have remained undetected. Second, the control group was not matched for all potential confounding variables, such as dietary habits, physical activity, or family history of CRC, which may influence the observed associations. Finally, the study focused exclusively on three selected SNPs in the *SIRT1* promoter region and did not account for other potentially relevant polymorphisms or gene–gene and gene–environment interactions that could modulate CRC risk. Despite these limitations, this study offers a solid foundation for further exploration of the role of *SIRT1* polymorphisms in colorectal cancer susceptibility and supports the growing relevance of personalized prevention strategies in oncology.

## 5. Conclusions

Although our findings did not reveal statistically significant differences in *SIRT1* gene polymorphism frequencies between the CRC group and the control group, the observed distribution may warrant further exploration in studies with greater statistical power. This research underscores the importance of understanding low-penetrance genetic factors in CRC, highlighting their potential to inform more personalized and effective prevention strategies.

## Figures and Tables

**Table 1:** Demographic characteristics of study groups and *SIRT1* genotyping with respect to cancer localization.

	1	2	3	4	5	*p*-Value 1 vs. 2	*p*-Value 1 vs. 3	*p*-Value 1 vs. 4	*p*-Value 1 vs. 5
	Controls (N = 120)	Rectal Cancer (N = 85)	Left-Side CRC (N = 65)	Right-Side CRC (N = 49)	CRC (All)				
sex						**0.0266**	0.1829	0.0859	0.0177
female	64 (53.3%)	32 (37.6%)	28 (43.1%)	19 (38.8%)	79 (39.7%)				
male	56 (46.7%)	53 (62.4%)	37 (56.9%)	30 (61.2%)	120 (60.3%)				
age						0.3506	0.1439	0.0045	0.0510
Mean (SD)	62.14 (11.67)	63.45 (8.31)	64.35 (8.59)	66.67 (8.06)	64.54 (8.40)				
BMI						0.1975	0.2595	0.9615	0.8504
N-Miss	2	0	1	0	1				
Mean (SD)	27.33 (4.56)	28.15 (4.38)	26.57 (4.15)	27.29 (4.59)	27.42 (4.39)				
*SIRT1*_rs12778366 *						0.6459	0.4095	0.2787	0.3302
TT	95 (79.2%)	65 (76.5%)	48 (73.8%)	35 (71.4%)	148 (74.4%)				
TC or CC	25 (20.8%)	20 (23.5%)	17 (26.2%)	14 (28.6%)	51 (25.6%)				
*SIRT1*_rs7895833 *						0.9068	0.4298	0.2584	0.4907
AA	80 (66.7%)	56 (65.9%)	47 (72.3%)	37 (75.5%)	140 (70.4%)				
AG or GG	40 (33.3%)	29 (34.1%)	18 (27.7%)	12 (24.5%)	59 (29.6%)				
*SIRT1*_rs3758391 *						0.5943	0.9934	0.4837	0.5922
CC	61 (50.8%)	40 (47.1%)	33 (50.8%)	22 (44.9%)	95 (47.7%)				
CT or TT	59 (49.2%)	45 (52.9%)	32 (49.2%)	27 (55.1%)	104 (52.3%)				

* Dominant model.

**Table 2:** *SIRT1* polymorphism and BMI in CRC vs. controls.

Control Group				CRC Group		
*SIRT1*_rs12778366				*SIRT1*_rs12778366		
	TT (N = 95)	TC or CC (N = 25)	*p*-value	TT (N = 148)	TC lub CC (N = 51)	*p*-value
BMI			0.1457			0.5733
N-Miss	2	0		1	0	
Mean (SD)	27.59 (4.79)	26.32 (3.51)		27.53 (4.42)	27.13 (4.33)	
*SIRT1*_rs7895833				*SIRT1*_rs7895833		
	AA (N = 80)	AG lub GG (N = 40)	*p*-value	AA (N = 140)	AG lub GG (N = 59)	*p*-value
BMI			0.268			0.9149
N-Miss	0	2		1	0	
Mean (SD)	27.64 (4.70)	26.67 (4.24)		27.40 (4.57)	27.47 (3.98)	
*SIRT1*_rs3758391				*SIRT1*_rs3758391		
	CC (N = 61)	CT lub TT (N = 59)	*p*-value	CC (N = 95)	CT lub TT (N = 104)	*p*-value
BMI			0.0327			0.747
N-Miss	0	2		1	0	
Mean (SD)	28.18 (5.07)	26.41 (3.78)		27.32 (4.78)	27.52 (4.03)	

**Table 3:** Association of *SIRT1* polymorphisms with CRC risk.

		Controls vs. Patients with CRC					
		Unadjusted			Adjusted for Sex, Age, and BMI		
		OR	95% CI	*p*-Value	OR	95% CI	*p*-Value
*SIRT1*_rs12778366	TT	Ref. level			Ref. level		
	TC or CC	1.31	0.76–2.25	0.3309	1.24	0.71–2.16	0.446
*SIRT1*_rs7895833	AA	Ref. level			Ref. level		
	AG or GG	0.84	0.52–1.37	0.4909	0.89	0.54–1.47	0.6473
*SIRT1*_rs3758391	CC	Ref. level			Ref. level		
	CT or TT	1.13	0.72–1.78	0.5923	1.14	0.72–1.82	0.5785

## Data Availability

The datasets used and/or analyzed during the current study are available from the corresponding author upon reasonable request.

## Supplementary Materials

The following supporting information can be downloaded at: https://www.mdpi.com/article/10.3390/jcm14113912/s1, Table S1: Distribution of *SIRT1* polymorphisms in female CRC patients and female controls; Table S2: Distribution of *SIRT1* polymorphisms in male CRC patients and male controls; Table S3. Association of *SIRT1* polymorphisms with Rectal Cancer risk; Table S4. Association of *SIRT1* polymorphisms with Left side CRC risk; Table S5. Association of *SIRT1* polymorphisms with Right side CRC risk.

## Abbreviations

The following abbreviations are used in this manuscript:

CRC	Colorectal cancer
*SIRT1*	Sirtuin 1
SNP	Single-nucleotide polymorphism
DNA	Deoxyribonucleic acid
qPCR	Quantitative polymerase chain reaction
HNPCC	Hereditary non-polyposis colorectal cancer
FAP	Familial adenomatous polyposis coli
HDAC1	Histone deacetylase 1
EDTA	Ethylenediaminetetraacetic acid
MAF	Minor allele frequency
OR	Odds ratio
BMI	Body mass index
FOXO	Forkhead box O
AKT	Serine/threonine-specific protein kinase
PDK1	Phosphoinositide-dependent protein kinase 1
DLBCL	Diffuse large B-cell lymphoma
