# Supplementary material for: Exploring the Role of SIRT1 Polymorphisms in Colorectal Cancer Risk: A Case–Control Study
Source: J Clin Med. 2025 Jun 2;14(11):3912. doi: 10.3390/jcm14113912 (PMC12156903; doi:10.3390/jcm14113912)
Supplement: Supplementary file 1 [file jcm-14-03912-s001.zip › jcm-3659797-supplementary.pdf]

Table S1. Distribution of *SIRT1* polymorphisms in female CRC patients and female controls.

		Controls vs. CRC patients					
		unadjusted			adjusted for age and BMI		
		OR	95% CI	p-value	OR	95% CI	p-value
SIRT1_rs12778366	TT	Ref. level			Ref. level		
	TC or CC	1.57	0.7-3.5	0.2710	1.58	0.71-3.54	0.2648
SIRT1_rs7895833	AA	Ref. level			Ref. level		
	AG or GG	0.79	0.39-1.62	0.5201	0.81	0.39-1.68	0.5762
SIRT1_rs3758391	CC	Ref. level			Ref. level		
	CT or TT	1.22	0.63-2.37	0.5504	1.23	0.63-2.4	0.5385

Table S2. Distribution of *SIRT1* polymorphisms in male CRC patients and male controls

		Controls vs. CRC patients					
		unadjusted			adjusted for age and BMI		
		OR	95% CI	p-value	OR	95% CI	p-value
SIRT1_rs12778366	TT	Ref. level			Ref. level		
	TC or CC	1.1	0.52-2.32	0.7974	0.98	0.45-2.1	0.9538
SIRT1_rs7895833	AA	Ref. level			Ref. level		
	AG or GG	0.87	0.44-1.71	0.6815	0.94	0.47-1.9	0.8640
SIRT1_rs3758391	CC	Ref. level			Ref. level		
	CT or TT	1.03	0.55-1.94	0.9296	1.04	0.54-2	0.9066

Table S3. Association of SIRT1 polymorphisms with Rectal Cancer risk

		Controls vs. Rectal CRC					
		unadjusted			adjusted for sex, age, BMI		
		OR	95% CI	p-value	OR	95% CI	p-value
SIRT1_rs12778366	TT	Ref. level			Ref. level		
	TC or CC	1.17	0.6-2.28	0.6461	1.17	0.59-2.32	0.6495
SIRT1_rs7895833	AA	Ref. level			Ref. level		
	AG or GG	1.04	0.58-1.86	0.9068	1.12	0.61-2.06	0.7041
SIRT1_rs3758391	CC	Ref. level			Ref. level		
	CT or TT	1.16	0.67-2.03	0.5944	1.25	0.71-2.23	0.437

Table S4. Association of SIRT1 polymorphisms with Left side CRC risk

		Controls vs. Left side CRC					
		unadjusted			adjusted for sex, age, BMI		
			95% CI	p-value	OR	95% CI	p-value
SIRT1_rs12778366	TT	Ref. level			Ref. level		
	TC or CC	1.17	0.6-2.28	0.6461	1.17	0.59-2.32	0.6495
SIRT1_rs7895833	AA	Ref. level			Ref. level		
	AG or GG	1.04	0.58-1.86	0.9068	1.12	0.61-2.06	0.7041
SIRT1_rs3758391	CC	Ref. level			Ref. level		
	CT or TT	1.16	0.67-2.03	0.5944	1.25	0.71-2.23	0.437

Table S5. Association of SIRT1 polymorphisms with Right side CRC risk

		Controls vs. Right side CRC					
		unadjusted			adjusted for sex, age, BMI		
			95% CI	p-value	OR	95% CI	p-value

SIRT1_rs12778366	TT	Ref. level			Ref. level		
	TC or CC	1.52	0.71-3.25	0.2805	1.32	0.6-2.92	0.4934
SIRT1_rs7895833	AA	Ref. level			Ref. level		
	AG or GG	0.65	0.31-1.38	0.2603	0.69	0.32-1.51	0.351
SIRT1_rs3758391	CC	Ref. level			Ref. level		
	CT or TT	1.27	0.65-2.47	0.4841	1.19	0.59-2.4	0.6194
